# New insights into glycoprotein Ibα desialylation-mediated platelet clearance

**DOI:** 10.1080/09537104.2020.1764922

**Published:** 2020-06-04

**Authors:** Jack Yule, Neil V. Morgan, Natalie S. Poulter

**Affiliations:** 1 Centre of Membrane Proteins and Receptors (COMPARE), Universities of Birmingham and Nottingham, Midlands, UK; 2 Institute of Cardiovascular Sciences, College of Medical and Dental Sciences, University of Birmingham, Edgbaston, UK

**Keywords:** Glycoprotein Ibα, O-glycans, platelet clearance, sialylation

Platelet count is tightly regulated in order to maintain an appropriate hemostatic response; too high and the risk of a thrombotic event increase; too low and bleeding occur due to an inadequate repair of the vascular injury site. Aside from hemostasis, a steady platelet count is also necessary for an appropriate immune response, due to their inflammatory role, both through secretion of cytokines and interactions with immune cells[1].

**Figure 1.**
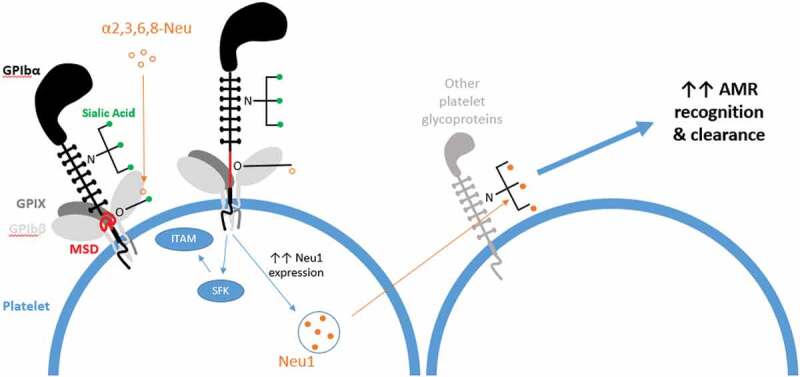
Sialic acid molecules (green) are present at the end of glycans that decorate GPIbα, a highly abundant platelet surface glycoprotein. Exogenous neuraminidase (e.g. α2,3 or α2,3,6,8-neuraminidase (open orange circles)) from invading bacteria removes sialic acid from O-glycans of the GPIbα mechanosensory domain (MSD, red), leading to its unfolding. This propagates signaling through GPIb-IX, leading to Src family kinase signaling and potentially subsequent immunoreceptor tyrosine-based activation motif (ITAM) signaling, which is similar to what occurs when VWF-A1 binds to GPIba[17]. MSD unfolding also induces expression and surface presentation of endogenous Neuraminidase 1 (Neu1; filled orange circles) found in platelet granules. This increases the desialylation of N-glycans on other platelet glycoproteins, which then preferentially bind the hepatic Ashwell-Morell receptor (AMR), allowing the platelet to be taken up and cleared.

A key challenge in this *‘balancing act’* is the relatively short platelet lifespan of 7–10 days in humans (3–5 days in mice), and the need for a reactionary approach to quickly replace platelets at the end of their lifecycle. With a normal platelet count in healthy individuals of 150-400x10⁹/L of blood, this amounts to approximately 10^11^ platelets being produced and destroyed per day.

To optimize this process, the major platelet production pathway, involving thrombopoietin (TPO) regulated bone marrow megakaryocyte proliferation and differentiation [2], is coupled to platelet clearance. The major pathway for clearance is regulated via binding of platelet surface glycoproteins to the Ashwell-Morell receptor (AMR), expressed primarily on hepatocytes [3], but it can also be achieved through binding to the myeloid-specific integrin αMβ2 (also known as MAC-1) on neutrophils and monocytes [4], or via pooling in the spleen. As platelets age, sialic acid is removed from glycans on the major surface glycoproteins, exposing galactose residues. Binding of AMR to these residues leads to clearance (via internalization and destruction of platelets), whilst simultaneously driving JAK2/STAT3 signaling, stimulating hepatic TPO production and therefore replenishing the platelet count[5].

GPIbα glycan desialylation is thought to be the major regulator of AMR-mediated clearance, given that it is one of the most highly expressed platelet surface glycoproteins and has a high level of glycosylation [6–8]. In support of this, previous work shows that its removal stunts the rate of clearance following conditions that lead to desialylation such as cold storage [9], ST3GalIV sialyltransferase knockout, or bacterial infections that release neuraminidase (sialidase) enzymes into circulation[3].

Whilst GPIbα N-linked glycans have previously been thought to be the major target in AMR-mediated clearance due to their tri- or tetra antennary arrangement being preferentially targeted by the AMR [10,11], *Wang et al., 2020* [12] uses the neuraminidase-induced mechanism of platelet desialylation to suggest that, during bacterial infections of this type at least, it is GPIbα mono-antennary O-glycans which are the key desialylation targets, that pave the way for enhanced clearance via subsequent desialylation of N-glycans.

This recent study involves the injection of neuraminidase derived from *Arthrobacter ureafaciens* into wildtype mice, which induces a sharp drop in platelet count from 24 hours that slowly recovers over 4–5 days whilst leaving red cell count unaffected, and is consistent with bacterial infections of this type[13]. They observe this same enhanced rate of clearance in both VWF global knockout, a ligand for GPIbα, and a megakaryocyte lineage-specific ADAM17 knockout, a sheddase targeting several membrane proteins, including GPIbα, suggesting they are not involved in this process. However, as there is substrate redundancy within the ADAM metalloprotease family, knocking out only ADAM17 is strong, but not conclusive evidence that there is no role for GPIba shedding in platelet clearance, whereas removal of GPIbα ectodomain (IL4 R-IbaTg: a chimeric construct where the GPIbα ectodomain is replaced with the α subunit of the Interleukin 4 receptor) stunts the increased clearance, maintaining a normal platelet count. Given the absence of this enhanced clearance in IL4 R-IbaTg mice, this supports previous literature which suggests that GPIbα is the major target of this mechanism due to its abundance, high level of glycosylation and therefore sialylation.

Furthermore, lectin binding assays indicate that both N- and O-linked glycans are desialylated following neuraminidase injection, both *in vitro* and *in vivo*. However, it is an O-linked glycan exposure specifically which correlates with the sharp drop in platelet count observed in mice, followed by a 4–5-day recovery. Crucially, specific N-glycan desialylation does resemble the drop-in platelet count at 24 h after treatment, indicating that there is some role for these in the enhanced clearance mechanism, and supports this paper’s conclusion that the O-glycan desialylation event is key to the induction of this clearance. Crucially, the fact that mouse GPIbα has no N-glycosylation sites yet demonstrates a drastic drop in platelet count, indicates that if GPIbα is indeed the major desialylation target, it is O-glycans that are the initial major target, as depicted in Figure 1.

There is however a decrease in platelet clearance in mice whose megakaryocytes and platelets are deficient in the sialyltransferase ST3Gal1 (St3gal1^MK-/-^), versus wildtype, despite the fact that this would likely cause a lower level of O-glycan sialylation and therefore should logically increase clearance. This could be a result of earlier GPIbα cleavage, and therefore would contribute to the proposed ‘enhanced’ clearance mechanism whereby O-glycan desialylation is an event that feeds back into N-glycan desialylation by GPIbα signaling and increased neuraminidase expression. Monitoring St3gal1^MK-/-^ platelet counts without neuraminidase treatment would have been beneficial to see if the baseline count is affected versus untreated wildtype mice and confirm this hypothesis.

The rationale behind this proposed mechanism of enhanced clearance centers on the effect that the removal of sialic acid has on the heavily O-glycosylated, juxtamembrane mechanosensory domain (MSD) of GPIbα, the unfolding of which was established by *Zhang et al., 2015*. A follow-up paper by Zhang et al. in 2019 established that the force required to unfold an unglycosylated MSD was significantly weaker than the physiological forces normally needed. This current study demonstrates that retention of glycans but the removal of sialic acid has a similar force-reducing effect, and in 76% of measurements no unfolding event was observed. Though this might point to a low level of unfolding it is consistent with prior models of an MSD which is natively unfolded and, perhaps surprisingly, occurs at the same frequency as in overstretching of unglycosylated MSD [14], suggesting a need for these modifications, in part or in entirety, for MSD stability.

In summary, this study by Wang et al helps to develop the emerging theory of MSD unfolding, following binding of GPIbα to its ligands such as VWF-A1 under physiological shear, and how this induces activatory signals through the GPIb-IX complex into the platelet, including the involvement of a signaling ‘Trigger’ sequence. GPIbα shedding is thought to be induced following signaling, supported in this study by the increased availability of the ADAM17 shedding site (housed within the MSD) following desialylation. This is worth clarifying in mouse platelets, as only those expressing human GPIbα were used.

A further implication of this exogenously induced desialylation and the resultant increase in GPIb-IX signaling would be the subsequent surface expression of endogenous neuraminidases, such as Neu1, as the platelet acts to mark senescent platelets for clearance following their activation. This would further increase desialylation, via the N-glycans on other platelet glycoproteins, thereby ‘enhancing’ clearance rate via the AMR.

Increased accessibility of the ADAM17 sheddase, plus ‘Trigger’ sequence exposure in IL4 R-IbaTg platelets would explain the mild thrombocytopenia in these mice and the apparent lack of ‘enhanced’ clearance upon neuraminidase treatment, as these platelets would be natively cleared very early on due to constitutive/elevated GPIb-IX signaling. The same could be said in St3gal1^MK-/-^ mice, explaining the relative decrease in clearance versus the wildtype. Going forward, it would be of use to repeat parts of this investigation using platelets obtained from patients with variants that may cause a lower native level of platelet glycoprotein sialylation, such as sialyltransferase mutants and that of reported patients with severe thrombocytopenia and mutations in the *GNE* gene, which is involved in sialic acid biosynthesis [15,16].

In conclusion, this work does not serve to replace the existing theory of N-glycan desialylation as the major AMR-mediated clearance target, even suggesting that N-glycans are involved within the first 24 h, when the majority of clearance occurs. In fact, this study proposes that this O-glycan targeting, ‘GPIb-facilitated clearance’ mechanism is a prerequisite to the enhanced platelet clearance observed in response to exogenous neuraminidase, introduced into circulation by invading bacteria.

## References

[1] Li R, Hoffmeister KM, Falet H. Glycans and the platelet life cycle. Platelets 2016 9;27(6):505–511. doi:10.3109/09537104.2016.1171304. Epub 2016 May 2. Review. [PubMed: 27135356] 27135356 PMC5396182

[2] Eaton DL, de Sauvage FJ. Thrombopoietin: the primary regulator of megakaryocytopoiesis and thrombopoiesis. Exp Hematol 1997;25(1):1–7. 8989900

[3] Sorensen AL, Rumjantseva V, Nayeb-Hashemi S, Clausen H, Hartwig JH, Wandall HH, Hoffmeister KM. Role of sialic acid for platelet life span: exposure of beta-galactose results in the rapid clearance of platelets from the circulation by asialoglycoprotein receptor-expressing liver macrophages and hepatocytes. Blood 2009;114(8):1645–1654. doi:10.1182/blood-2009-01-199414. PubMed: 19520807. 19520807 PMC2731641

[4] Ross GD. Regulation of the adhesion versus cytotoxic functions of the Mac-1/CR3/alphaMbeta2-integrin glycoprotein. Crit Rev Immunol 2000;20:197–222. doi:10.1615/CritRevImmunol.v20.i3.20 10968371

[5] Grozovsky R, Begonja AJ, Liu K, Visner G, Hartwig JH, Falet H, Hoffmeister KM. The Ashwell-Morell receptor regulates hepatic thrombopoietin production via JAK2-STAT3 signaling. Nat Med 2015;21(1):47–54. doi:10.1038/nm.3770. PubMed: 25485912. 25485912 PMC4303234

[6] Berndt MC, Gregory C, Kabral A, Zola H, Fournier D, Castaldi PA. Purification and preliminary characterization of the glycoprotein Ib complex in the human platelet membrane. Eur J Biochem 1985;151(3):637–649. doi:10.1111/j.1432-1033.1985.tb09152.x. PubMed: 3161731. 3161731

[7] Zhang W, Deng W, Zhou L, Xu Y, Yang W, Liang X, Wang Y, Kulman JD, Zhang XF, Li R, et al. Identification of a juxtamembrane mechanosensitive domain in the platelet mechanosensor glycoprotein Ib-IX complex. Blood 2015;125(3):562–569. doi:10.1182/blood-2014-07-589507. PubMed: 25359992. 25359992 PMC4296016

[8] Gröttum KA, Solum NO. Congenital thrombocytopenia with giant platelets: a defect in the platelet membrane. Br J Haematol 1969;16(3):277–290. doi:10.1111/j.1365-2141.1969.tb00402.x. PubMed: 4893927. 4893927

[9] Rumjantseva V, Grewal PK, Wandall HH, Josefsson EC, Sørensen AL, Larson G, Marth JD, Hartwig JH, Hoffmeister KM. Dual roles for hepatic lectin receptors in the clearance of chilled platelets. Nat Med 2009;15(11):1273–1280. doi:10.1038/nm.2030 19783995 PMC4428152

[10] Lodish HF. Recognition of complex oligosaccharides by the multi-subunit asialoglycoprotein receptor. Trends Biochem Sci 1991;16(10):374–377. doi:10.1016/0968-0004(91)90154-N 1785139

[11] Spiess M. The asialoglycoprotein receptor: a model for endocytic transport receptors. Biochemistry 1990;29(43):10009–10018. doi:10.1021/bi00495a001 2125488

[12] Wang Y, Chen W, Zhang W, Lee-Sundlov MM, Casari C, Berndt MC, Lanza F, Bergmeier W, Hoffmeister KM, Zhang XF, Li R. Desialylation of O-glycans on glycoprotein Ib a drives receptor signaling and platelet clearance. Haematologica 2020;105:xxx. doi:10.3324/haematol.2019.240440 PMC7776245 31974202

[13] Grewal PK, Aziz PV, Uchiyama S, Rubio GR, Lardone RD, Le D, Varki NM, Nizet V, Marth JD. Inducing host protection in pneumococcal sepsis by preactivation of the Ashwell-Morell receptor. Proc Natl Acad Sci U S A 2013;110(50):20218–20223. doi:10.1073/pnas.1313905110 24284176 PMC3864324

[14] Zhang XF, Zhang W, Quach ME, Deng W, Li R. Force-regulated refolding of the mechanosensory domain in the platelet glycoprotein Ib-IX complex. Biophys J 2019;116(10):1960–1969. doi:10.1016/j.bpj.2019.03.037 31030883 PMC6531785

[15] Futterer J, Dalby A, Lowe GC, Johnson B, Simpson MA, Motwani J, Williams M, Watson SP, Morgan NV. Mutation in GNE is associated with severe congenital thrombocytopenia. Blood 2018;132(17):1855–1858. doi:10.1182/blood-2018-04-847798 29941673 PMC6238157

[16] Revel-Vilk S, Shai E, Turro E, Jahshan N, Hi-Am E, Spectre G, Daum H, Kalish Y, Althaus K, Greinacher A, et al. GNE variants causing autosomal recessive macrothrombocytopenia without associated muscle wasting. Blood 2018;132(17):1851–1854. doi:10.1182/blood-2018-04-845545 30171045 PMC6202914

[17] Gardiner EE, Arthur JF, Shen Y, Karunakaran D, Moore LA, Am Esch JS, Andrews RK, Berndt MC. GPIbα-selective activation of platelets induces platelet signaling events comparable to GPVI activation events. Platelets 2010;21(4):244–252. doi:10.3109/09537101003695339 20367574

